# An Individual Patient's “Body” on Chips—How Organismoid Theory Can Translate Into Your Personal Precision Therapy Approach

**DOI:** 10.3389/fmed.2021.728866

**Published:** 2021-09-13

**Authors:** Uwe Marx, Enrico Accastelli, Rhiannon David, Hendrik Erfurth, Leopold Koenig, Roland Lauster, Anja Patricia Ramme, Petra Reinke, Hans-Dieter Volk, Annika Winter, Eva-Maria Dehne

**Affiliations:** ^1^Department of Medical Biotechnology, Institute of Biotechnology, Technische Universität Berlin, Berlin, Germany; ^2^TissUse GmbH, Berlin, Germany; ^3^Functional and Mechanistic Safety, Clinical Pharmacology & Safety Sciences, R&D, AstraZeneca, Cambridge, United Kingdom; ^4^Berlin Center for Advanced Therapies, Charité-Universitätsmedizin Berlin, Berlin, Germany; ^5^BIH-Center for Regenerative Therapies, Berlin Institute of Health, Charité-Universitätsmedizin Berlin, Berlin, Germany

**Keywords:** organismoid, organ-on-chip, microphysiological systems, real world data, immune-oncology, advanced therapies, organoid, patient-on-chip

## Abstract

The first concepts for reproducing human systemic organismal biology *in vitro* were developed over 12 years ago. Such concepts, then called human- or body-on-a-chip, claimed that microphysiological systems would become the relevant technology platform emulating the physiology and morphology of human organisms at the smallest biologically acceptable scale *in vitro* and, therefore, would enable the selection of personalized therapies for any patient at unprecedented precision. Meanwhile, the first human organoids—stem cell-derived complex three-dimensional organ models that expand and self-organize *in vitro*—have proven that *in vitro* self-assembly of minute premature human organ-like structures is feasible, once the respective stimuli of ontogenesis are provided to human stem cells. Such premature organoids can precisely reflect a number of distinct physiological and pathophysiological features of their respective counterparts in the human body. We now develop the human-on-a-chip concepts of the past into an organismoid theory. We describe the current concept and principles to create a series of organismoids—minute, mindless and emotion-free physiological *in vitro* equivalents of an individual's mature human body—by an artificially short process of morphogenetic self-assembly mimicking an individual's ontogenesis from egg cell to sexually mature organism. Subsequently, we provide the concept and principles to maintain such an individual's set of organismoids at a self-sustained functional healthy homeostasis over very long time frames *in vitro*. Principles how to perturb a subset of healthy organismoids by means of the natural or artificial induction of diseases are enrolled to emulate an individual's disease process. Finally, we discuss using such series of healthy and perturbed organismoids in predictively selecting, scheduling and dosing an individual patient's personalized therapy or medicine precisely. The potential impact of the organismoid theory on our healthcare system generally and the rapid adoption of disruptive personalized T-cell therapies particularly is highlighted.

## Introduction to the Organismoid Theory

**Figure 1:**
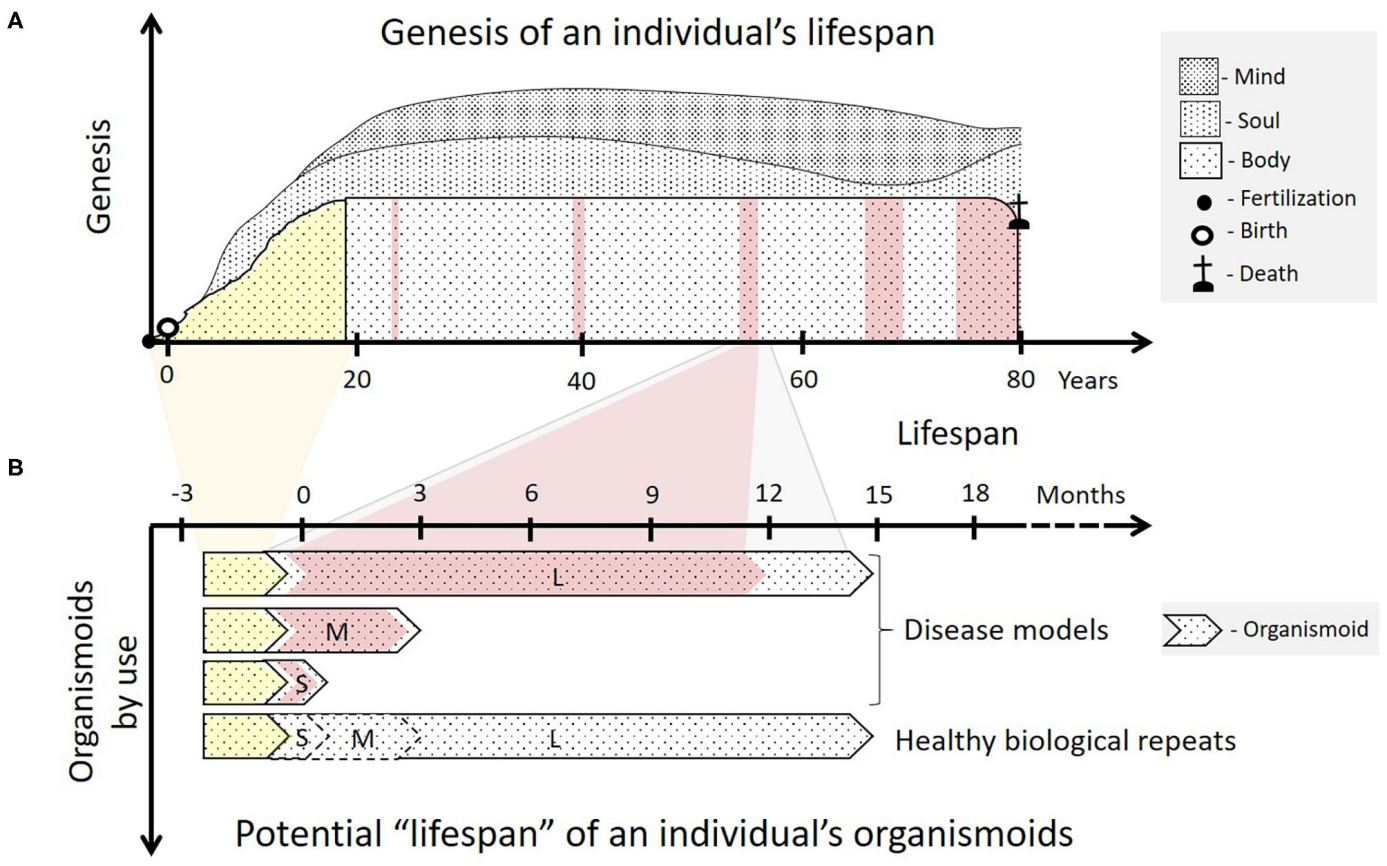
Organismoids in the context of each human individual's fate. **(A)** The ontogeny (yellow) of an individual's body begins with ovum fertilization, followed by birth and ends with sexual maturity, a fully functional brain and an adult skeleton after 18 to 20 years. The adult body then goes through a lifelong period of relatively functional and architectural homeostasis lasting many decades. This adulthood is interrupted with increasing frequency by periods of ever-prolonging illness and recovery as the body ages (pink). Emotions and consciousness—the soul and the mind of a human being—begin to develop consecutively in childhood and continue to do so throughout life (sociogenesis). **(B)** According to the organismoid theory, personalized organismoids can be established through accelerated *in vitro* ontogenesis (yellow) lasting a few months. The resulting adult organismoids can then emulate a certain stage of healthy human adulthood for weeks (S—short-term), months (M—mid-term) or years (L—long-term), depending on use. These can then be utilized to emulate acute, sub-chronic and chronic disease periods (pink) and therapy-based recovery of an individual within the respective time frame. A large number of identical organismoids ensure that a sufficient number of healthy biological repeats can be run simultaneously serving as controls for full recovery of the diseased organismoids by a precision medicine or advanced therapy approach. Moreover, such healthy organismoids are useful to evaluate preventive medicine approaches, such as vaccination for the respective individual.

A human individual's lifespan is characterized by phases of development (ontogenesis) and functional maintenance (adulthood) of the physiology and morphology of the human body and a lifelong sociogenesis of an individual's soul and mind in a bidirectional person to population context (1), schematically illustrated in Figure 1A.

Sociogenesis is linked intrinsically to the morphological size and architecture of the human brain defined—consisting of around 86 billion neurons and a roughly equal number of non-neuronal cells (2) that are highly interconnected and clustered to process, integrate and coordinate the information it receives from the sense organs (3)—and its interconnections with the rest of the body. The physiology of the mature human body follows a simple evolutionary, selected building plan where form follows function. Back in 2007, we drew attention to the fact “[…] that almost all organs and systems are built up by multiple, identical, functionally self-reliant, structural units [...] ranging from several cell layers to a few millimeters. Due to distinguished functionality, a high degree of self-reliance and multiplicity of such structural units within the respective organ, their reactivity pattern to drugs and biologics seem representative of the whole organ. Nature created these small, but sophisticated, biological structures to realize most prominent functions of organs and systems. The multiplication of these structures within a given organ is Nature's risk-management tool to prevent the total loss of functionality during partial organ damage. In evolutionary terms, however, this concept has allowed the easy adjustment of organ size and shape to the needs of a given species (e.g., liver in mice and men), while still using almost the same master plan […]” (4). In 2012, this knowledge, combined with progress in the development of microphysiological systems (MPS), provided the basis for the first conceptual visions of emulating human bodies at the smallest biologically acceptable scale on biochips (5–7). At that time, we introduced the concept of a “man-on-a-chip” at a downscale factor of 100,000. We illustrated the functional units of the major human organs and briefly described the downscale principle (5). This was the starting point for developing a theory of the establishment of minute mindless and emotion-free physiological *in vitro* equivalents of an individual's human body, which we now call organismoids. Different terminologies, such as human-on-a-chip, body-on-a-chip, or universal physiological template, have been used in the past for organismoids, but it is common sense among the MPS community that the targeted organismal homeostasis can be achieved by combining the prime organ equivalents from at least the following 10 human systems: circulatory, endocrine, gastrointestinal, immune, integumentary, musculoskeletal, nervous, reproductive, respiratory and urinary. A chip-based system interconnecting these organ models will compose a minimal organismal equivalent and the MPS community forecasts at least another decade to establish such functional organismoids on chips (8, 9).

**Figure 2:**
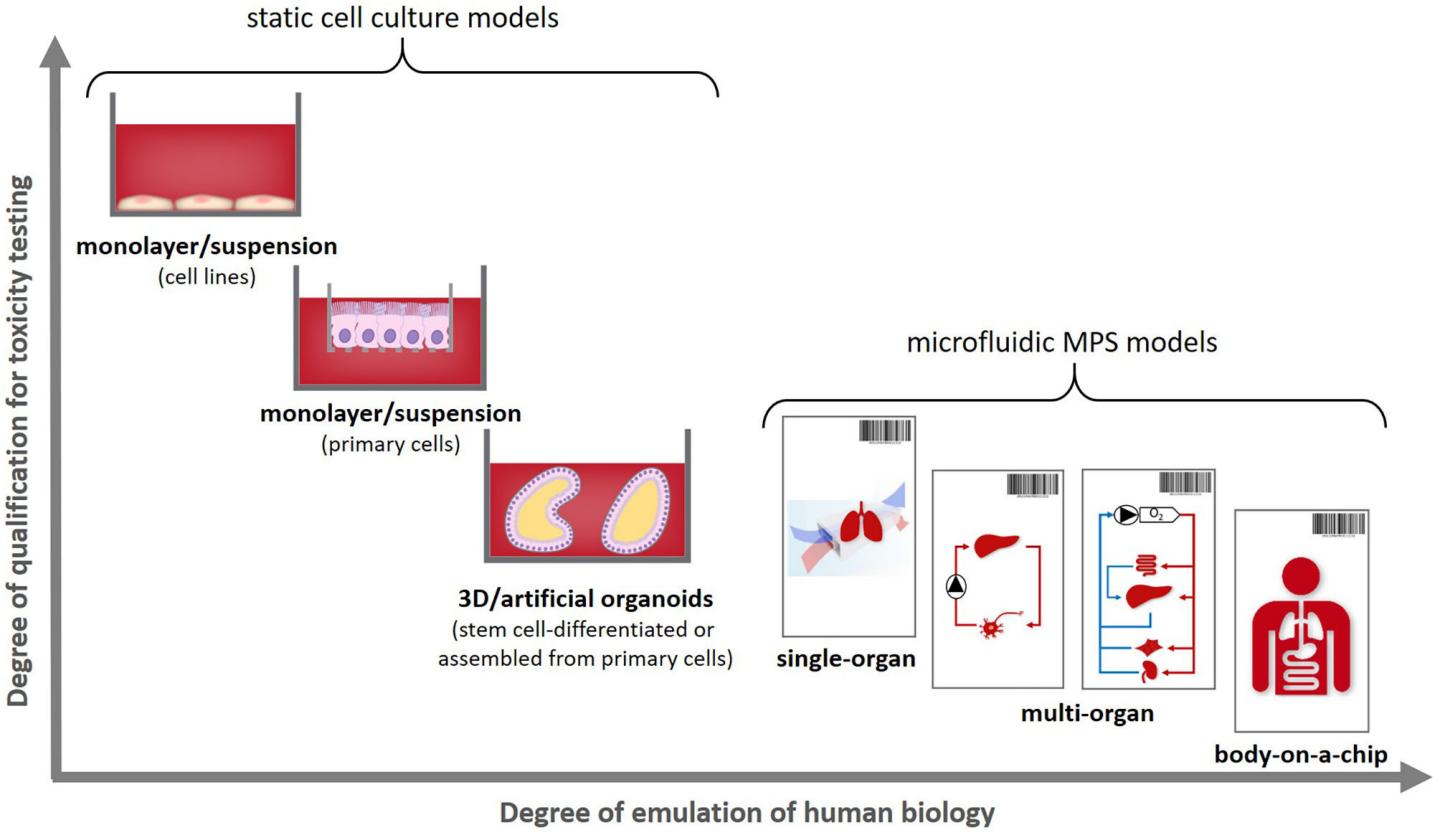
Positioning human organismoids—body-on-a-chip equivalents—within the current cell model landscape [adopted from (10)].

These can be used to emulate an individual patient's disease and healthy state, as illustrated in Figure 1B, therewith enabling a precise selection of the right medicine or therapy and the most efficacious exposure regime for each patient. In addition to this use for precision medicine approaches organismoids from selected cohorts of patients can further be used to conduct clinical trials on chips. Their position within the current landscape of cell models regarding their potential to emulate human physiology was illustrated in 2018 by the Investigative Toxicology Leaders Forum, which brought together representatives from 14 European pharmaceutical companies (Figure 2) (10).

The organismoid theory is based on two chronologically interrelated concepts, each with three principles for implementation. The concept of *in vitro* ontogenesis of an individual's organismoids relies on the principles of (i) (induced pluripotent) stem cell-based formation of premature organoids of an individual body *in vitro*; (ii) physiology-based integration of the relevant type and ratio/numbers of such premature organoids into premature self-sustained organismoids through whole blood perfusion and innervation, applying on-chip MPS; and the (iii) completion of *in vitro* ontogenesis toward healthy mature organismoids (emulating the adult stage) by organoid on-chip cross talk and accelerated exposure to ontogenic stimuli.

Subsequently, the concept of emulating the process of disease and healing of an individual patient using his/her organismoids on chips follows the principles of (i) induction of a disease in organismoids by natural disease processes or the transmission of pathogens or diseased tissues derived from the patient; (ii) the mimicry of a human clinical trial with a large number of patients by a trial with the equivalent number of healthy and diseased organismoids of one single patient; and (iii) the precise selection of the right medicine or therapy and the most efficacious exposure regime for each individual patient.

In this paper, we take you through the concepts and principles of organismoid theory, underpin the most important aspects with actual results and observations, describe its disruptive potential for our healthcare system and provide an outlook on possible approaches to a final proof of the theory.

## What did Stem Cell- and Patient-Derived Organoids Teach Us?

Deciphering the biochemical and biophysical cues leading to tissue-specific morphogenesis and organogenesis *in vivo* has fascinated scientists for over a century. Their studies were confined to classical cell culture and animal models for lack of more physiologically relevant test systems. These models have vastly enhanced our basic understanding of cellular function and disease mechanisms. However, translation of results to the human situation has become a major bottleneck. Recent advancements in the field of stem cell research and three-dimensional (3D) culture systems have led to the generation of a promising complex and completely human model system called organoids. These organoids are generated from either pluripotent stem cells (PSCs) (either induced PSCs [iPSCs] or embryonic stem cells), adult stem cells (ASCs) or adult tumor tissue by self-organization. Their use for drug discovery and personalized medicine has been reviewed (11) and first proof of concept to generate personalized data has been provided (12).

Organoid tissues maintain their capacity to keep proliferating and differentiating into the different cell types of the respective organ, while preserving a stem cell pool, by carefully tuning microenvironmental cues, such as mimicking the *in vivo* stem cell niche. In comparison to two-dimensional monolayer cultures, 3D organoid cultures mimic more closely the physiological behavior of organs shown by gene and protein expression and metabolization capacity. The majority of organoids require an extracellular matrix environment, which is based on laminins and collagen, comparable to the physical scaffold surrounding cells *in vivo*. This matrix is, in most cases, animal-based and not well-defined, therefore, batch-to-batch variabilities might occur (13). Furthermore, the differentiation process of organoids, ASC- or PSC-derived, depends on many different elements, such as growth factors, the matrix, matrix stiffness, cell-cell contact, cell density, oxygen level, nutrient supply or the stochastic nature of *in vitro* self-organization and cell fate decision. Thus, there is a high heterogeneity in the maturation and function of organoids under standard *in vitro* culture conditions.

Organoids derived from PSCs mimic embryonic development *in vitro*. Therewith, these organoids are of great value for developmental studies. Different growth factors are used to push the PSCs into the appropriate germ layer—mesoderm, endoderm, or ectoderm. Subsequently, further growth factor cocktails are used to drive the cells to form a differentiated organoid. Here, matrix proteins play a crucial role in organoid formation and are frequently used to mimic the basal lamina. The differentiated organoids may consist of different cells types—epithelial and mesenchymal—and may even acquire initial endothelial networks by the intrinsic differentiation (14) or extrinsic addition of endothelial cells or mesodermal progenitors (15, 16). The period of generating PSC-derived organoids varies depending on the tissue type and usually requires between 2 weeks and 3 months (17) but can continue for half a year or longer, as seen in skin (18) or brain organoids (19).

Patient-specific PSC-derived organoids are generated by reprogramming somatic cells into iPSCs. However, this may take several months. The efficiency of organoid differentiation varies greatly between tissue types and even differentiation protocols. Furthermore, there is a limited possibility for the passaging of iPSC-derived organoids.

Organoids are difficult to generate from ASCs for some tissues, such as the brain, due to a lack of availability of tissue samples. Therefore, PSC-derived organoids are beneficial for brain organoid generation. These organoids have been cultured for up to and beyond 2 years in 3D spheroid suspension culture without passaging (19, 20).

Organoids derived from ASCs are generated from adult tissue having regenerative ability. Early human tooth or hair follicle development models, for example, apply mesenchymal condensation principles *in vitro* to generate the placode or the dermal papilla organoids, respectively from donor-derived progenitor cells (21, 22). However, only the epithelial portion of tissues can be made into organoids. Stromal cells, endothelial cells and nerves are missing in these models. The major benefits of ASC-derived organoids lie in the fact that they may be generated from healthy or tumor tissue. Organoid formation of ASCs normally takes only several days and the organoids are stable for long-term cultivation and expansion. Distinct growth factor cocktails are used for organoid expansion and differentiation, therefore, they can be expanded indefinitely. Biobanks of healthy and tumor organoids from patients can be generated from different organs to test drugs in high throughput screenings for further decision-making for patient treatment (17).

A large variety of human organoids has been generated using these 3D cultivation methods in academic labs over the past decade. Their applications and their potentials have been extensively reviewed (13, 17, 23–25). Table 1 highlights the human organs for which organoids have been generated in static *in vitro* culture. However, conventional static culture systems cannot terminally differentiate the organoids into mature and fully functional organ models. Local morphogen gradients are appearing in organoids as they are forming but stable blood perfusion-driven morphogen gradients of growth factors, oxygen and other functional biochemical or biophysical cues are missing. Thus, static culture conditions limit the cultivation time due to restrictions in the supply of nutrients and waste removal from ever-growing organoids, therewith limiting their maturation grade. In the following, we will discuss how to improve organoid maturity by introducing defined spatiotemporal cues.

**Table 1:** Overview of human organoids generated in static *in vitro* culture.

**Organ**	**Tissue source**	**Culture condition**	**Functionality or application**	**References**
Blood vessel	iPSCs, epithelial stem cells (ESCs)	Spheroids in ultralow attachment plates or matrix-embedded	Endothelial cells and pericytes that self-assemble into capillary networks	(26)
Brain	iPSCs, ESCs	Suspension spheroids or matrix-embedded	Unpatterned organoids contain cell clusters with forebrain, midbrain, hindbrain and retinal identities that contain glutamatergic, GABAergic and dopaminergic neurons as well as astroglia. Patterned organoids can be differentiated toward forebrain, midbrain, brainstem, cerebellum, thalamus, hypothalamus, spinal cord and hippocampus identity	(19, 27–42)
Liver	ASCs, ESCs, iPSCs	Suspension spheroids or matrix-embedded	Appropriate secretion ability (albumin and urea) and drug metabolic ability (CYP3A4 activity and inducibility)	(43–56)
Thyroid	Adult thyroid-derived cells	Matrix-embedded	Secretion of thyroid hormones	(57)
Pancreas	ASCs, ESCs, iPSCs	Matrix-embedded	Secretion of insulin in response to glucose	(58–60)
Optic cup	iPSCs, ESCs	Matrix-embedded	Primitive cornea and lens-like cells, developing photoreceptors, retinal pigment epithelia, axon-like projections and electrically active neuronal networks	(61–66)
Intestine	ASCs, ESCs, iPSCs	Matrix-embedded	Villus- and crypt-like structures and enterocytes, goblet, enteroendocrine, and Paneth cells	(67–71)
Gastric	ASCs, iPSCs	Matrix-embedded	Used to study H pylori infection and other gastric pathologies	(72–75)
Kidney	ASCs, ESCs, iPSCs	Spheroids or matrix-embedded	Rudimentary nephrons, and 3D culture combined with active fluid flow	(76–81)
Lung	ESCs, iPSCs, fetal lung tissue, ASCs	Matrix-embedded	Rudimentary bronchiole-like structures and express alveolar cell markers	(82–87)
Skin	iPSCs	Aggregates in suspension with matrix coating, skin Transwell model	Complex skin analogs with human iPSC (hiPSC)-derived keratinocytes, endothelial cells and fibroblasts	(18, 88, 89)
Cardio	iPSCs, ESCs	Spheroids or monolayer	Contractile spheroids	(90–94)
Mammary gland	adult mammary gland tissue	In adherent or floating matrix	Could be induced to produce milk protein	(95, 96)
Prostate	ASCs	Matrix-embedded	With basal and luminal cells	(97–99)

## Microfluidic Cell Culture Systems—the Key Toward the Integration of Premature Organoids Into Organismoids

Organoids have proven to be powerful tools in emulating distinct sets of organ-specific characteristics. However, marker expression and functionality often halts at a premature stage, as described above. We have known since 1912 that the environment of *in vitro* cultures defines their viability and functionality (100). The isotropic microenvironmental cues that have driven organoid self-assembly and differentiation engulf organoids under traditional culture conditions rather homogenously or cover extensive surface areas hindering the spatial orientation and maturation driven by functionality. But these spatiotemporal cues originating from interacting tissues and leading to a rearrangement of cells are key to the development of mature organ functionality. Endothelial-tissue cross talk in particular and its implications for local signaling during organogenesis have been studied extensively (101–103). Vascularization of the developing central nervous system, for example, is a crucial step in brain development ensuring oxygen and nutrient supply of the rapidly dividing neural progenitors. Neural structures of the peripheral nervous system have been demonstrated to develop in noticeable alignment with blood vessels. Furthermore, the importance of endothelial cells for the maintenance of the germinal zones of the central nervous system where cerebellar cells are produced has been shown (104).

Moreover, the recombinant proteins and small molecules administered in static cultures to control lineage specification mostly promote the development of a specific subsection of cells within the organ (the parenchymal cells). Other crucial lineages, such as vascular, neuronal or immune lineages, are mostly absent, therewith silencing paracrine signaling that might become relevant during further maturation.

Allowing a finely orchestrated systemic interaction of premature organoids with other organ systems at physiologically relevant scales promotes an alignment of functionality and a higher spatial resolution of stimulation. Nature's organ building blocks—the smallest functional units described above—form, in their multiplicity, an entire human organ. The number of those repetitive subunits depends on the requirements signaled by the interacting organs. Therewith, organ sizes, medium flow rates and fluid residence times in organs and overall liquid to cell ratios self-adjust in a dynamic interplay of tissues.

Several approaches enabling the systemic interaction of tissue models have been devised to date—the most prominent being patient-derived xenograft (PDX) models and MPS. In the former, immunodeficient or humanized mice serve as hosts, enabling the engraftment of primarily tumor models. The interplay of grafts with local and systemic environments, also through a vascularization of the models, eventually allows for a nutrition of cells and a propagation of models. However, species' differences between the host organism and the patient's tissue prevents a complete match of biology. A plethora of drawbacks have been described in using these methods, but the assets of having a systemic circulation supporting the models could be shown.

Substantial efforts have been made over the past two decades to improve organ model culture conditions by introducing them into MPS. Dozens of human organ equivalents in MPS have been established using primary- and cell line-based models and have been reviewed in great detail (105–111). It is well-documented that the maturation of organ function can be achieved by closely emulating organotypic microenvironments regarding biochemical, physical, or electrical stimuli (106).

Once the notion became clear that the autologous nature of such systems is essential, the MPS community started to establish stem cell-derived models on-chip. Table 2 summarizes the very recent achievements in this area. Furthermore, it is only a matter of time before the missing organoids for the creation of minimal organismoids are established.

**Table 2:** Examples of the MPS-based models established recapitulating functions of the key human organs.

**Organ**	**Substructure/Cell types**	**Tissue source**	**(Patho-) Physiology**	**Chip type**	**MPS advantage**	**References**
Brain	Unpatterned (forebrain + hindbrain)	hiPSCs	Healthy, + prenatal nicotine exposure	Continuous unidirectional perfusion	Enhanced expression of cortical layer markers (TBR1 and CTIP2) under perfusion	(112, 113)
	Unpatterned (forebrain + midbrain + hindbrain)	hESCs	Healthy	Continuous unidirectional perfusion	Creation of signaling gradients that mimic developmental patterning for neural tube formation	(114)
	Blood-brain barrier (BBB; endothelial cells + motor neurons)	iPSCs	Healthy	Continuous unidirectional perfusion	Increased calcium transient function and chip-specific gene expression under perfusion	(115)
	BBB (endothelial cells + neural progenitor)	iPSCs	Healthy + diseased (Huntington's disease)	Continuous unidirectional perfusion	Physiologically relevant TEER and BBB permeability, capillary wall protected neural cells from plasma-induced toxicity	(116)
	GABAergic neurons and astrocytes	iPSCs	Healthy	Continuous bidirectional perfusion		(117)
Optic Cup	Retina	hiPSCs	Healthy	Continuous unidirectional perfusion	Recapitulation of the interaction of mature photoreceptors with retinal pigment epithelium	(118)
Liver	Hepato- and cholangiocytes	hiPSCs	Healthy	Continuous unidirectional perfusion	Improved cell viability, higher expression of endodermal mature hepatic genes and improved functionality under perfusion	(119)
	Hepatocytes	hiPSCs	Healthy	Continuous unidirectional perfusion	Higher potential hepatic progenitor cells to hepatic organoids under perfusion	(120)
Pancreas	Islet-specific α- and β-like cells	hiPSCs	Healthy	Continuous unidirectional perfusion	Enhanced expression of pancreatic β-cell gene and protein expression and increased β-cell hormone production under perfusion	(121)
Heart	Cardio-myocytes	hiPSCs	Healthy + diseased (Barth syndrome)	Continuous unidirectional perfusion	Enabled description of metabolic, structural and functional abnormalities associated with Barth syndrome	(122)
	Cardio-myocytes + endothelial cells	hiPSCs	Healthy	Continuous unidirectional perfusion	Endothelial cells align with the flow and form tube-like networks in the cardiac muscle channel	(123)
Intestine	Duodenum	Human ASCs (tissue biopsies)	Healthy	Continuous unidirectional perfusion	Human-relevant functionality is superior to that of organoids alone	(124)
	Unpatterned	hiPSCs	Healthy	Continuous unidirectional perfusion	Polarized, contains all the intestinal epithelial subtypes and is biologically responsive to exogenous stimuli	(125)
	Small intestine	Human ASCs (tissue biopsies)	Healthy	Repeated unidirectional perfusion	Removal of dead cells from the organoid tubes under perfusion allowed long-term culture > 1 month	(126)
	Unpatterned	hiPSCs	Healthy	Continuous unidirectional perfusion	Luminal waste removal through continuous flow	(127)
Stomach	Gastric organoids	hiPSCs	Healthy	Continuous closed loop perfusion	Rhythmical stretch and contraction—reminiscent of gastric motility	(128)
Kidney	Glomerolus (podocytes)	hiPSCs	Healthy	Continuous unidirectional perfusion	Differential clearance of albumin and inulin when co-cultured with human glomerularendothelial cells	(129)
	Glomerolus + tubulus organoid	hiPSCs	Healthy	Continuous unidirectional perfusion	Generation of perfusable vascular networksand better cell maturation under perfusion	(130)
Multi-organ	Brain, intestine, kidney, liver	hiPSCs	Healthy	Continuous circular perfusion	Co-culture over 14 days in one common medium deprived of tissue-specific growth factors	(131)

A huge variety of additional human tissue and organ models have been published. However, attempts at organismal on-chip homeostasis have so far failed to integrate the systemic components, such as whole blood supply through vascularized microvessels, a personalized (autologous) immune system and tissue innervation. Here, we have extended the theorization of creating MPS-based organismoids by integrating the premature organoids of each relevant organ system into a self-regulating vascularized and innervated systemic circulation.

We hypothesized already in 2012 that “the lack of a dynamic interplay between organ-specific cell types, with their vascular and stromal tissue bed, and the absence of adult stem cell and progenitor niches for local regeneration, are responsible for the crucial missing capabilities of current ‘human-on-a-chip’ systems” (5). The vascularization of whole microfluidic circuits on-chip was shown as early as 2013 (132), followed by fascinating work on the generation of vascularized organ models on-chip (133–136). As of today, high-throughput platforms generating vascularized single-tissue models on-chip are commercially available (137). The combination of both technologies generating a closed vascularized circuit containing multiple organs on-chip is within reach, allowing for the next level of physiological complexity—the perfusion of whole blood or a defined substitute containing all relevant components.

**Figure 3:**
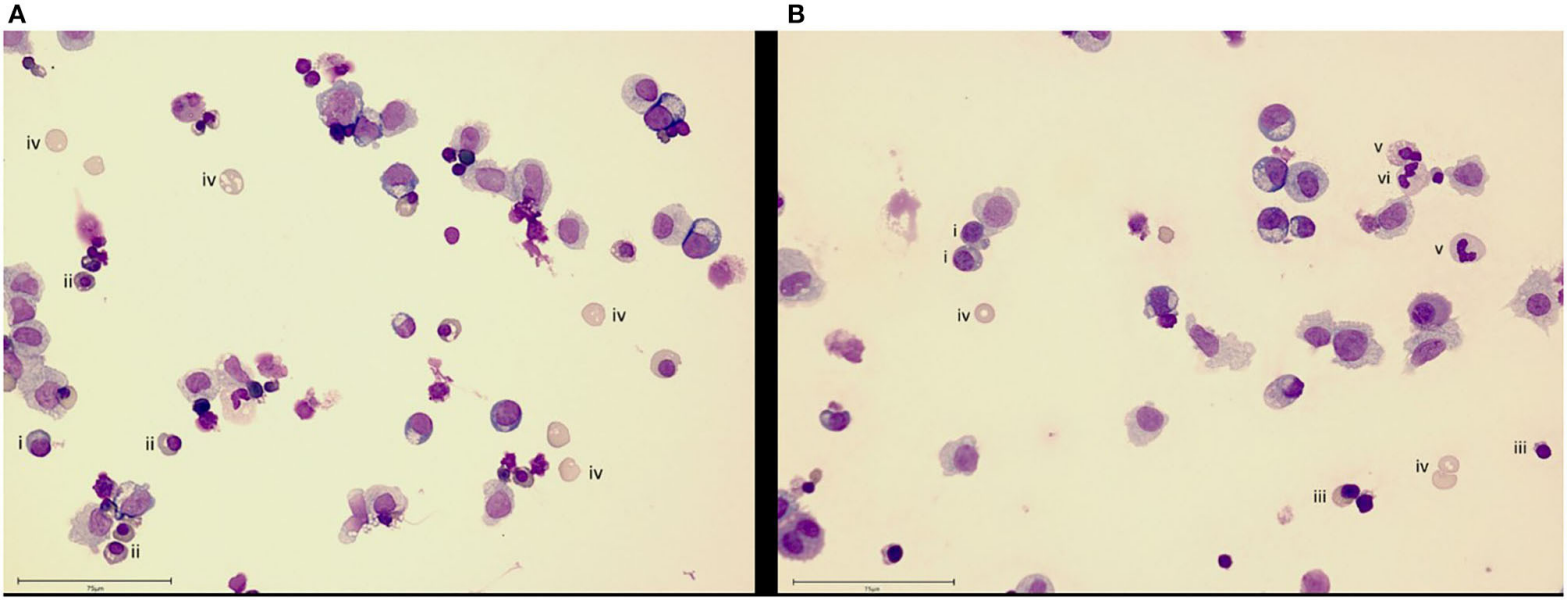
Bone marrow cell maintenance and differentiation in an MPS. Culture of human CD34+ cells on a human mesenchymal stem cell-seeded scaffold in the recirculating HUMIMIC Chip2 for **(A)** 17 or **(B)** 24 days shows differentiation into erythroid (day 17) and then, additionally, neutrophil (day 24) lineage cells. Cells are identified as (i) basophilic normoblast, (ii) polychromatic normoblast, (iii) orthochromatic normoblast, (iv) reticulocyte, (v) band cell and (vi) neutrophil. Scale bar−75 μm.

The organismoid theory hypothesizes that the generation and renewal of all crucial whole blood components—red blood cells, platelets, white blood cells and plasma components—is feasible and will lead to a self-sustained systemic organismoid. Therefore, a steady functional on-chip hematopoiesis is required. Several approaches to model the human bone marrow-based hematopoietic stem cell niche using MPS have been described (8, 138, 139). Adapting the model of Sieber et al. (138) to include cytokines important for cell differentiation and stem cell maintenance has enabled the continuous, robust generation and maintenance of cells from erythroid, myeloid and megakaryocyte lineages, while simultaneously maintaining stem and progenitor cell populations for at least 24 days (Figure 3). In brief, bone marrow chips were established as described by Sieber et al. (138) but with the modification of media to include additional cytokines, as outlined in Chou et al. (139). Cells were sampled from the recirculating media and deposited directly onto slides using a cytospin centrifuge, before staining with Wright's stain and imaging. The donor information for the cells used in the study is detailed in Table 3.

**Table 3:** Stem cell donor information for the bone marrow MPS.

**Human Mesenchymal Stem Cells (MSC)**				**Human CD34+** **Hematopoietic Stem Cells (HSC)**			
**Donor (Lot #)**	**Sex**	**Age**	**Race**	**Donor (Lot #)**	**Sex**	**Age**	**Race**
0000451491	Male	25	Caucasian	0000680575	Female	21	Black

The academic MPS development landscape has provided a number of other indicators that particular elements of blood perfusion can be recapitulated using MPS. The capability of emulating platelet-induced blood coagulation has been demonstrated by Westein et al. (140) and numerous publications describe the circulation of immune cells in MPS and their settlement in organ models on chips (141–143).

**Figure 4:**
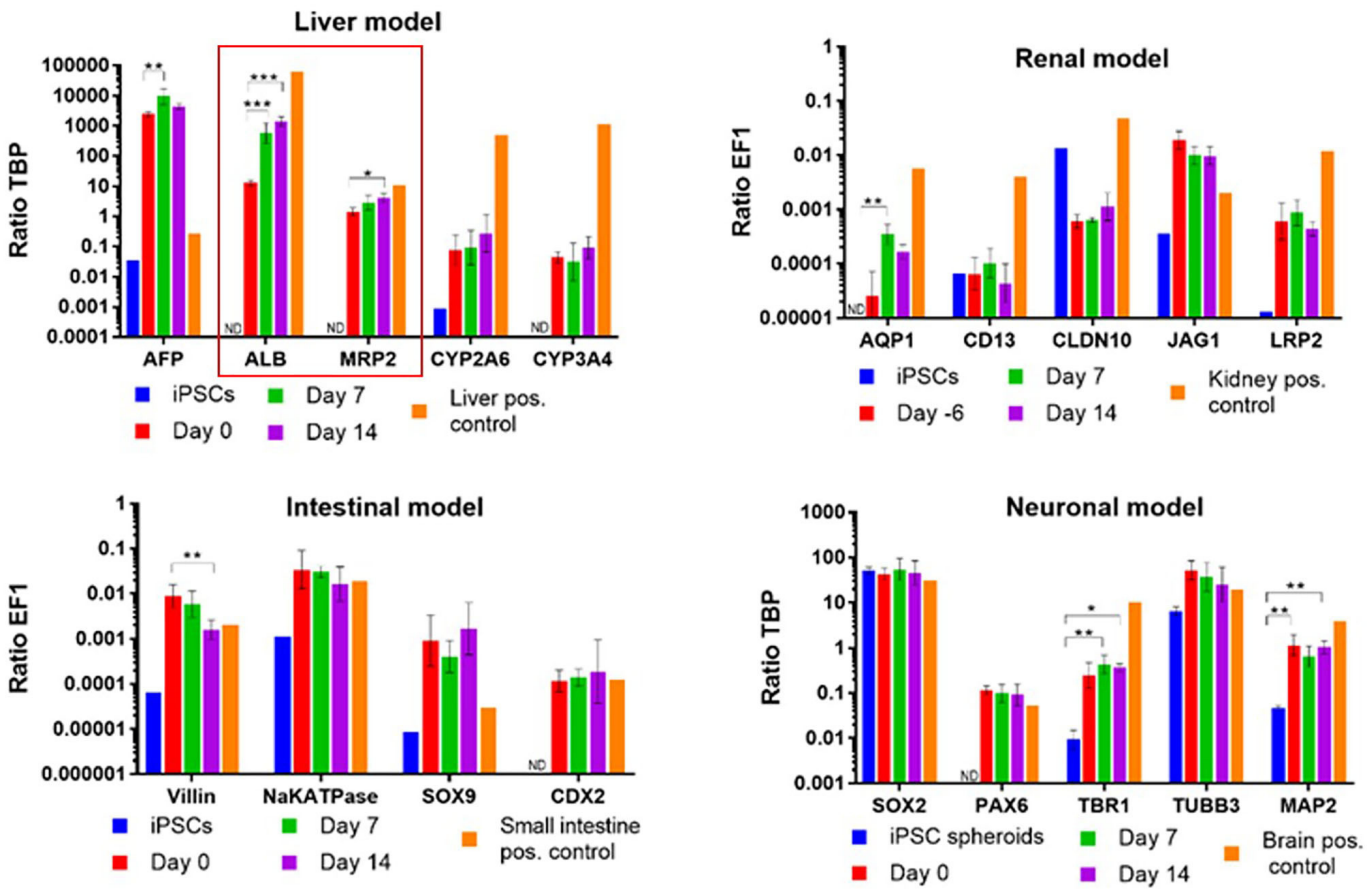
Albumin and MDR2 gene expression increased steadily from day 0 to 14 in a 4-organ chip composed of iPSC-derived premature intestinal, liver, kidney, and neuronal organ models integrated in a physiology-based common media circulation. Feeding of differentiation factor-free medium solely through the intestinal model was performed. One-way ANOVA with Tukey's multiple comparisons test was used for statistical analysis (*p < 0.05, **p < 0.01, ***p < 0.001). Data shown as Mean + SD.

As soon as it comes to organismoid-based physiological whole blood provision to all on-chip organ equivalents, the assets of using stem cell-derived organoids of an autologous source becomes relevant to prevent foreign organ model rejection by the immune system. Multi-organ systems published previously were mostly composed of tissues from different donors, which made the rejection-free integration of an individual's immune system, as the major defense mechanisms of any human organism, impossible. The first steps toward an autologous co-culture of several cell types from one iPSC donor were reported as early as 2013 (144). The premature nature of iPSC-derived organoids raised the question of how such organoids can be finally differentiated to match the functionality of their respective human counterparts. Here, the organismoid theory proposes the principle of terminal on-chip differentiation, guided by integrated organismal cross talk and artificially accelerated “training programs” for key organs and systems, such as xenobiotic panel exposure for the liver, multi-antigen vaccination for the immune system or artificial exposure to steroid hormones for the accelerated maturation of the sexual organs. The first hint that further on-chip maturation is triggered by organ-organ interaction in a physiology-based 4-organ chip was demonstrated in 2019 (131), where the expression of albumin and MRP2 genes increased significantly over a period of 14 days in an iPSC-derived premature liver model, driven solely by differentiation factor-free co-culture with iPSC-derived intestinal, kidney and neuronal models (Figure 4).

Such data support the organismoid theory's concept that once liver functionality matches the requirements of the systemic organismoid, a regular application of nutrients through the intestinal model and removal of waste substances through the kidney model will suffice to maintain functional homeostasis of the organismoid.

The majority of plasma proteins in humans are produced by the liver with albumin at a concentration of about 40 g/L, plasma being by far the largest component. In addition, the liver is a gatekeeper for toxicants and xenobiotics arriving through the food. Finally, it keeps homeostatic conditions regarding protein, carbohydrate and amino acid metabolism throughout the circulation. A plethora of MPS literature has provided evidence that human liver equivalents on chips composed of primary or iPSC-derived hepatocytes are capable of continuously secreting albumin and other proteins into the circulation (119). Another important protein component is the immunoglobulin G fraction produced by white blood plasma cells, reaching physiological levels of 7–16 g/L in the plasma protein fraction in humans. Very few MPS have aimed to generate immunoglobulins in modeling immune tissues, but an artificial lymph node has made initial progress here (145). Some other organs add crucial regulatory proteins to the plasma. Insulin secreted by the pancreatic islets and interacting with the liver to control the body's glucose-based energy balance through the regulation of glucose consumption, storage and release is such a key regulator. A stable pancreatic islet–liver co-culture MPS demonstrating the capability of an MPS to physiologically manage the secretion and organ interaction capacity for insulin was established in 2017 (146). Regarding plasma properties, it still remains a challenge to increase the plasma protein concentration generated on-chip toward physiological levels of 60–80 g/L, which is a factor of outstanding importance to leverage the physiological transport properties of albumin, deliver the hundreds of other functional proteins to their sites of action, and provide the right viscosity and flow dynamics for the transport of blood cells across the organism. The complete separation of the organ models from the blood flow by endothelial cells is a basic prerequisite for this and will provide progress in that field in the near future.

A preliminary adjustment of the plasma composition by technical means is feasible by closely monitoring metabolic activities on-chip by online sensors and might lead to an advanced maturation of organ models in the long term. Here, the use of automated systems maintaining a close observation of on-chip cultures will become an essential component (147).

Sensors can generally play a crucial role in the implementation of the organismoid theory, extending an individual's data generation beyond any currently available. On the one hand, the sensors can be inherent in the system. Analogous to pulse oximetry, for example, the oxygen saturation in the blood of an organismoid can be measured using different optical spectra of the various hemoglobin derivatives. In addition, pulse oximetry provides qualitative information about the pulsatile properties of the blood (148). The measurement of the oxygen saturation can be performed using miniaturized sensors or spatially resolved by using hyperspectral imaging techniques. The measurement of the oxygen saturation provides important information about the oxygen transport capacity, oxygen distribution and, in combination with microparticle imaging velocimetry analysis, the absolute oxygen consumption rate of each organoid incorporated into an organismoid (149).

On the other hand, the sensors can be embedded directly into the MPS. In parallel with the body area network technology for the health monitoring of the human body (150), the integration of multiple sensors is fundamental to allow the continuous online monitoring of organ-specific reactions and dynamic tissue responses. Multi-sensor integrated platforms are especially important for the development of MPS-based organismoids in which the monitoring of function of various organs requires a combination of different sensing principles. Transepithelial electrical resistance measurement is among the most popular non-invasive techniques and has been successfully integrated into MPS to assess the barrier integrity and junction dynamics of endothelial or epithelial models (151). Electrical impedance spectroscopy-based methods overcome conventional transepithelial electrical resistance measurement techniques by exploiting extended frequency domain data, thus, allowing an evaluation of tissue barrier function at different maturation stages (152). Impedance-based techniques can be further enhanced when coupled with multi-electrode arrays to provide localized sensing and electrical stimulation in relevant microenvironments of an organismoid. Applications include the recapitulation of cardiomyocyte or motor neuron innervation by direct electrical stimulation of the contractile activity (153), and the possibility of producing surrogate electroencephalograms from neuronal activities, which represents an added value to Parkinson or Alzheimer's disease modeling.

The innervation of organ models plays a pivotal role in their development, maturation, regulatory control, regeneration and pathology. We know from organ transplant surgeries that the ablation of autonomic neuronal connections may cause poor graft functionality and detrimental health effects (154). Similarly, the importance of innervation in the regulation of stem cells and/or their niches in most organs and tissues has been well-documented. Autonomic nerves impact tissue growth during the initial organogenesis and regeneration and, similarly, impact aging or the development and progression of disease. The introduction of innervation to *in vitro* models has so far been mostly neglected due to the complexity of achieving proper guidance and integration of neurons into non-neural tissue models. First steps have been taken to model both the synaptic junction between neural models and the neuroeffector junction formation between neural and non-neural tissue models both in static and MPS organoid cultures during the last decade. A major challenge in this field is the directed guidance of axon growth from the neuronal to the effector tissue. Consequently, the development of MPS that enable the assembly and cultivation of stem cell-derived myelinated motor neurons, as published by (155, 156), are an important basis for enabling functional innervation in MPS. Understanding how tissue-derived neurotrophic and neural guidance factors drive axon growth and determine its directionality during development will be instrumental.

Stem cell-derived regionalized brain organoids have been shown to possess the intrinsic capability of forming synaptic innervations with each other. Multiple groups have described the fusion of regionalized stem cell-derived cortical and subpallium organoids to model the migration of GABAergic interneurons from the subpallium organoids into the glutamatergic excitatory, neuron-rich cortical organoids, where they integrate functionally into local excitatory circuits (157–159). This intrinsic capability can also be exploited for the assembly of the other regionalized brain organoids, as has been shown for stem cell-derived cortical and thalamic organoids (159).

Roger D. Kamm's group has shown that stem cell-derived motor neuron organoids can build functional neuromuscular junctions (NMJs) with 3D skeletal muscle bundles in a patterned chip platform which enhanced the guided innervation of tissue constructs. Motor neurons in this setup were transduced with the light-sensitive channel rhodopsin-2, which enabled light-activated muscle contraction to show the formation of functional NMJs (160). Another study with stem cell-derived cerebral organoids showed that they could develop pronounced axon tracts, which functionally innervated into rodent spinal cord explants where they caused concerted muscle contractions easily distinguishable from local spontaneous contractions and could be evoked by electrical stimulation (31). The positive effects of nerve innervation on the maturity and functionally of cardiac tissue was shown by (161), where the innervation of sympathetic neurons increased the spontaneous beat rate of primary cardiac cells.

Protocols have been developed to omit the complex guidance of axon growth to the respective non-neural tissue organoid; these allowed the simultaneous differentiation of neural and non-neural tissue in one organoid. This has been achieved for neuromuscular organoids that contain functional NMJs and myelinated axons in the presence of terminal Schwann cells and contractile activity of the muscle part, which stopped upon blockage of acetylcholine receptors (162). Such interorgan spheroids were also realized for a combination of forebrain and optic vesicles, where bilateral light-sensitive optic vesicles developed on the surface of forebrain organoids and formed electrically active primitive sensory circuits (62).

The next big steps in the field of *in vitro* tissue innervation will be to build a closed neuronal circuit model with a sensory (e.g., optic cup organoid) and effector (e.g., muscle fiber) that are interconnected by a cortical model, and achieving the myelination of motor neurons axons by Schwann cells or oligodendrocytes (156, 163). Another promising approach is the combination of on-chip vascularization and innervation. *In vivo* peripheral nerves grow along blood vessels. We hypothesize that this route of innervation will also become relevant on-chip once the closed vascular system is established. The advantages of the interconnection of vascularization and innervation were shown by (116), who demonstrated vascular-neural interaction leading to a more *in vivo*-like gene expression signature and increased calcium transient in a MPS equipped with stem cell-derived brain microvascular endothelial cells and motor neurons.

In addition to the positive effects of physiological tissue innervation by (motor-)neurons, modeling of the peripheral nervous system is also of interest for the field of neurodegenerative diseases such as amyotrophic lateral sclerosis. *In vitro* (MPS) models have the potential to become an important cornerstone for studying the pharmacological effects of compounds on the NMJs. A first step in this direction was recently published by (164) on the Mimetas OrganoPlate platform, which hosts 40 microchips with iPSC-derived motor neurons. The latter showed pronounced axon outgrowth and could be coupled to muscle tissue to form NMJs.

Another critical aspect regarding organismal homeostasis is the interaction of autonomic innervation with the immune system. Looking at the digestive tract, for example, the gut immune system impacts on the local enteric nervous system, the extrinsic neurons of sympathetic and parasympathetic systems and, ultimately, on brain functions, such as mood, cognition and mental health. Conversely, the brain is able to modulate immune function in the intestine through the vagus nerve via the intestinal cholinergic anti-inflammatory pathway (165). Innervation in primary lymphoid organs, such as the bone marrow, and secondary lymphoid organs, such as the spleen, has been well-studied and the ability of the nervous system to influence immune homeostasis and inflammation in these niches has been shown (166).

Another important aspect to emulate systemic organismal pathways on chips includes the integration of the relevant donor-specific microbiota to mimic a patient's interaction with the respective metabolites in general (167–169).

It appears that innervation, vascularization, lymphatics, microbiota, and the emulation of a human-like enterohepatic circulation of bile products are indispensable prerequisites to bridge the gap between the simple physical combination of organoids in multi-organ MPS and real tissue interaction and homeostasis in an organismoid.

The latter needs a biological combination of the prime organ equivalents from at least 10 human systems (as highlighted in the introduction) and their biological interconnection through vasculature, innervation and lymphatics. Two early attempts to establish MPS containing at least 10 technically interconnectable organ culture compartments have already been published. Those prime examples include the 13-organ culture compartment system of the Shuler Lab at Cornell University (170) and the 10-organ culture compartment PhysioMimix™ system of the Griffith lab at MIT (171). Both systems have been successfully operated with biological materials in the culture compartments for seven or more days. However, both lack a biological blood vessel interconnection, lymphatics and organ innervation.

## What Organismoids Might Deliver to Our Healthcare System

According to the organismoid theory, organismoids are biological replica of the living human body *in vitro*, reduced in scale as far as possible. They are created by the systemic physiological integration of the functional units of the major human organs into an organismal, self-sustained template that reflects the systemic organization of the human body. The on-chip fast-track differentiation of stem cell derived organ equivalents originates from their cross talk and a physiological reliance on each other. The extreme reduction in scale is due to the goal of generating a large number of replicates of the organismoid of an individual. Large numbers of such identical, minute, mindless and emotion-free physiological *in vitro* equivalents of an individual's mature human body can be maintained at self-sustained functional healthy homeostasis over very lengthy time frames. They are open to perturbation leading to the natural or artificial induction of diseases. The diseased organismoids are hypothesized to emulate the pathophysiology of the respective patient's disease precisely. This, in turn, may enable the performance of predictive patient-specific organismoid studies to determine the most effective personalized therapy for the patient concerned. Similar to clinical studies on patient cohorts, statistically verified predictions can then be generated with the advantage that genetically identical replicates of the patient's organismoids can be compared under physiological and pathophysiological conditions. Two major usage scenarios can be derived from that. One is related to a cutting-edge improvement of an individual patient's personal treatment in the real world; the other has the potential to change the drug development paradigm on a clinical trial level, saving enormous amounts of time and capital expenditure.

**Figure 5:**
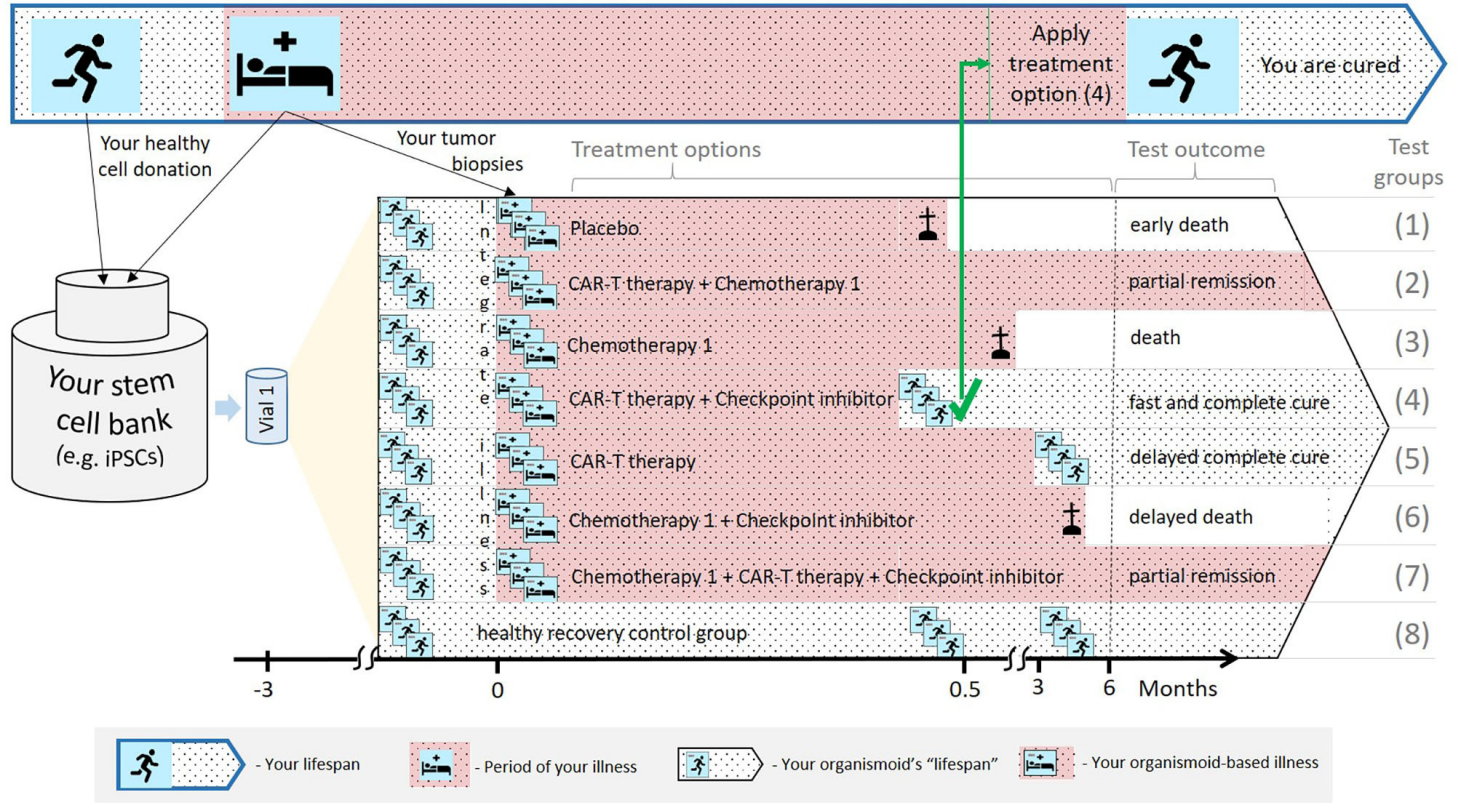
A hypothetical example illustrating how the organismoid theory can be utilized in personalized medicine.

Regarding the first scenario, organismoids can be used in predictively selecting, scheduling and dosing an individual patient's personalized therapy or medicine accurately along their disease progression. This can significantly decrease the potential risk to each and every patient by the early detection of unsuccessful treatment schedules. Figure 5 summarizes the advantages of applying organismoids for personalized precision medicine in more detail. The figure illustrates the concept and principles of the organismoid approach to select the best fitting precision medicine applied to your personalized illness. As a hypothetical example, cancer is chosen to be the illness.

Your lifespan might eventually include periods of life-threatening illness, for example, cancer growth (top: Blue-bordered arrow). A pluripotent stem cell bank is established from your healthy cells. Subsequently, a large number of identical healthy organismoids are generated within a few months (yellow triangle). Various options are currently available to treat cancer, therefore, relevant test groups are created, including placebo treatments, other treatment groups and healthy recovery controls (in the black-bordered arrow). In this hypothetical example, within a few weeks, the CAR-T cell therapy in combination with a checkpoint inhibitor turns out to be the fastest and most effective cure for you. Therefore, this therapy is immediately and successfully applied.

According to the organismoid theory, an individual's stem cell bank can be created when healthy or from a healthy organ when illness occurs. Preventive stem cell banking (e.g., from umbilical cord blood) is already in use and will be the choice in the future as it takes time. The near-to-human element of the theory provides precise test results which animal tests in patient-derived xenograft models or human patient-derived organoids cannot achieve. Xenograft models are phylogenetically distant and, therefore, cannot provide sufficient tumor outgrowth. Additionally, they do not have a patient's immune background to fight cancer. Patient-derived organoids are also not embedded in the patient's immune system and lack systemic interaction with the organism.

Regarding the second scenario, the average success rate of drug candidates entering clinical trials to become an approved drug has been below 20% for decades; no other industry can afford this inefficiency of translating any prototype into a marketed product. Poor predictivity of the preclinical safety and efficacy evaluation program of candidate drugs using laboratory animals is the prime reason for that inefficiency. Lengthy clinical trials averaging 13.5 years and bearing cumulative costs reaching as much as 2.5 billion USD to get a new medicine approved are the consequences (106). Simultaneously, a revolution in therapeutic strategies has emerged over the last three decades based on biologics—using the human body's own tools to fight diseases. The expanding biological complexity of medicines, from synthetic low molecular weight drugs toward, for example, human monoclonal antibody proteins and, finally, patient-specific autologous cell therapies, has dramatically increased the chances of cure for patients in recent years. However, this trend has, just as dramatically, reduced the chances of being able to predict the safety and efficacy of such therapies by applying preclinical laboratory animal testing due to the increasingly human origin of such advanced therapeutic medicinal products (172). In addition, the pricing along this rising biological gradient of medicines has become the major roadblock for the socially equitable availability of such therapies for all patients in the last few years. At the beginning of that trend, the average daily dose of a biologic drug cost 22 times more than that of a small molecule and accounted for a few dozen USD (173). However, best in class protein biologics—monoclonal antibodies—reached an annual average price for a patient's therapy course of about 96,000 USD in 2017 (174), which corresponds to roughly 263 USD per day. Nowadays, the price for the most disruptive innovation in advanced cell therapies—highly effective autologous CAR-T cell therapies—in Germany, for example, rose to as much as 320,000 € for a patient's treatment, considering a payment “at” result (175). This therapy is a single day infusion. An ever growing misbalance between the efficacy of wonder-performing drugs and the patient's financial ability to access them has become a serious social and economic conflict for our healthcare systems on a global scale.

**Figure 6:**
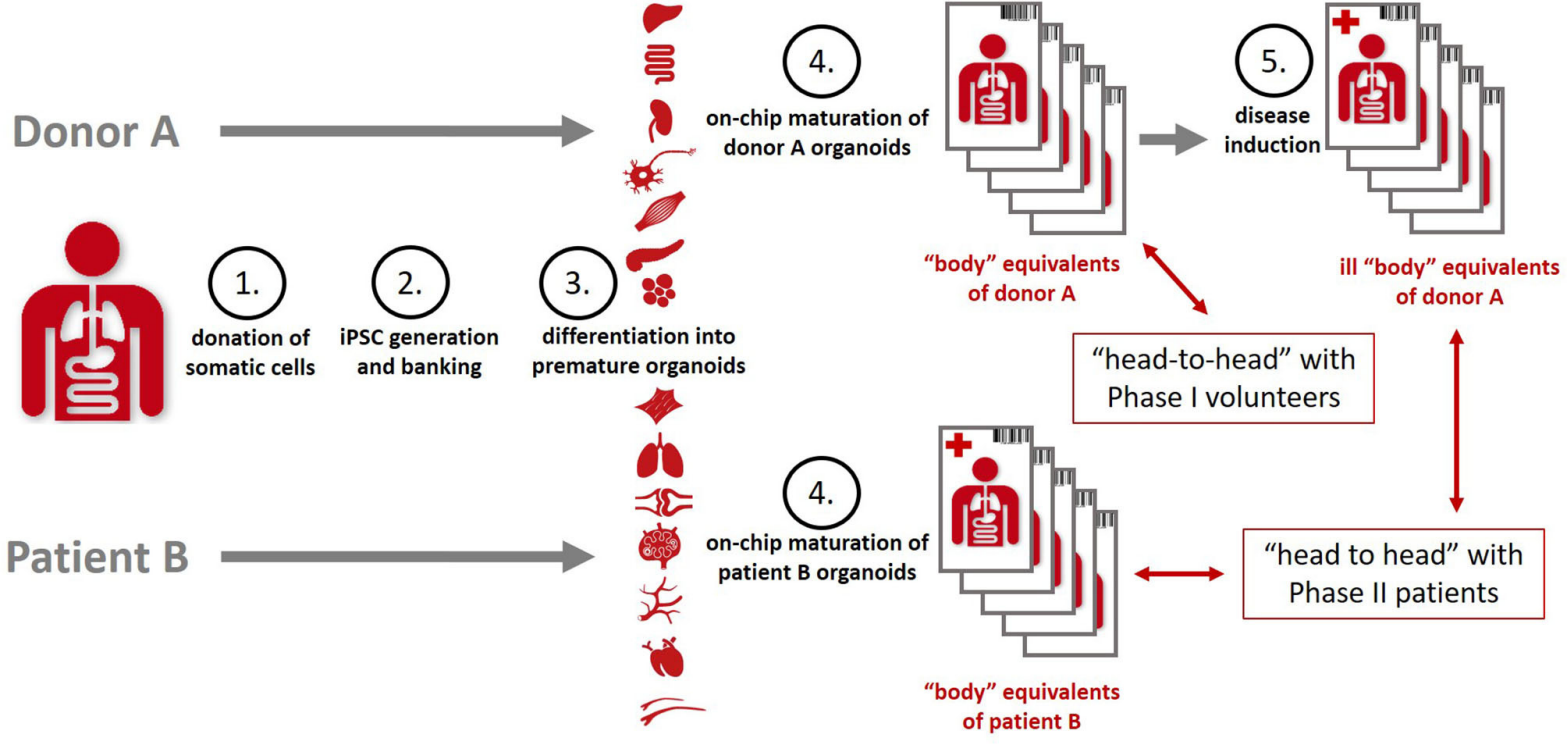
Organismoids—“body” equivalents—in the context of potential “clinical trials” on chips [adopted from (10)].

Organismoids have the potential to break this cost spiral by bringing about a paradigm shift in drug development. The stakeholder report of the MPS community produced in 2016 already projected a decrease of the cumulative drug development costs by a factor of five and a halving of the drug development times once MPS-based clinical trial-like studies on organismoids have enabled the accurate prediction of efficacy, safety, dosing and scheduling for any new medicine or therapy prior to use in human and replacing animal testing and Phase 1 and 2 clinical trials (106). An advanced roadmap toward the qualification of the precision of prediction of “clinical trials” (Figure 6) running minute personalized “body” equivalents (organismoids) in on-chip studies head-to-head with clinical trials was sketched in 2018 by the Investigative Toxicology Leaders Forum (10).

**Figure 7:**
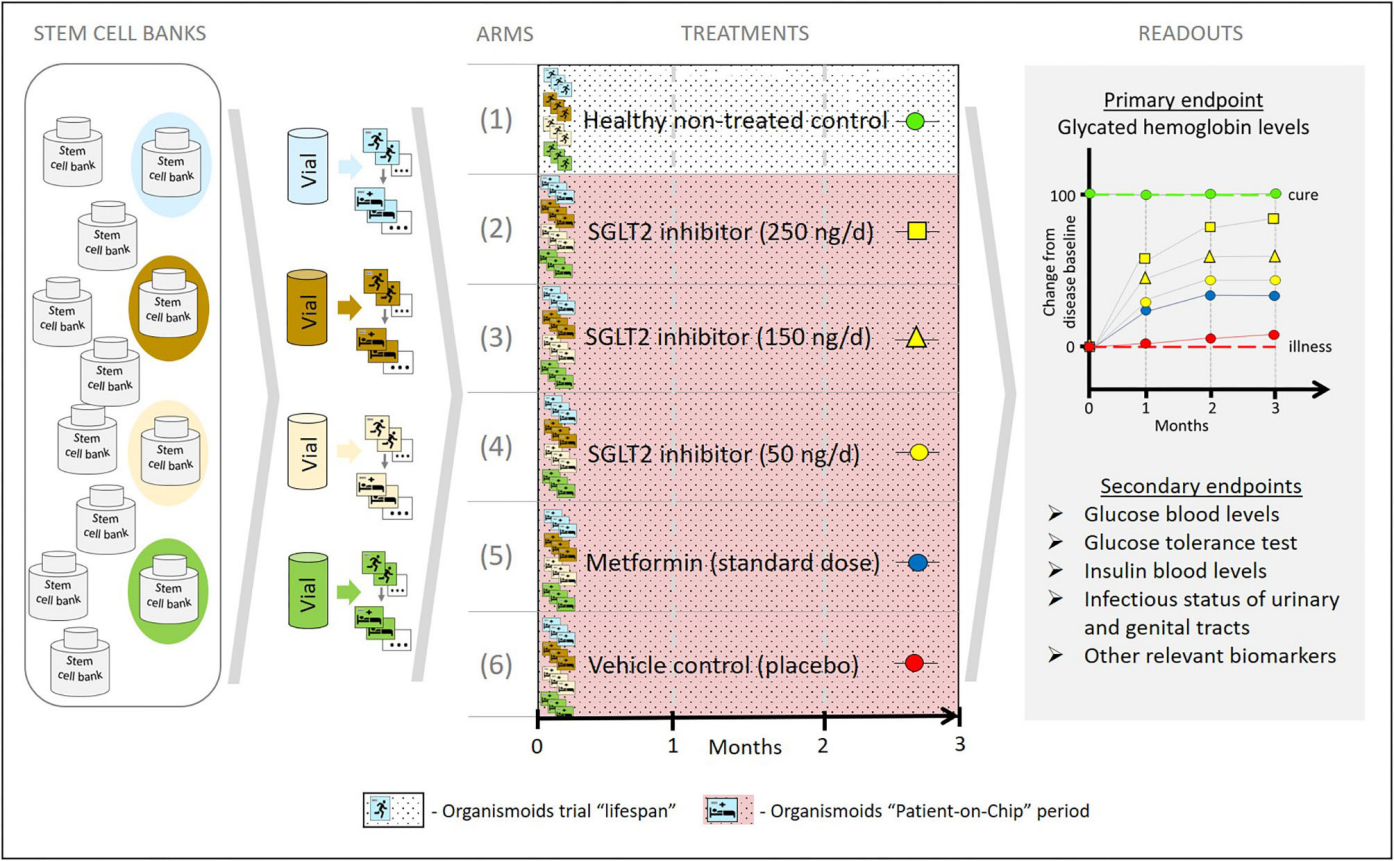
A hypothetical example illustrating how the organismoid theory can be utilized to emulate clinical trials.

In order to achieve that, a set of healthy and diseased organismoids representing the patient's disease status and their healthy homeostasis would allow one to conduct organismoid-based preclinical serial testing of medicines and advanced therapies in a setting emulating clinical trials with large trial-specific patient cohorts. In contrast to trials with patient cohorts, organismoid-based trials offer a number of crucial advantages. Figure 7 details these advantages by illustrating a hypothetical example of emulating a clinical trial of a hypothetical new sodium glucose transporter 2 (SGLT2) inhibitor to treat diabetes Type 2 utilizing an organismoid-based trial.

Stem cell samples from four donors representing a relevant diabetes patient cohort are collected from a global stem cell bank pool according to criteria equivalent to those of a clinical trial recruitment. A relevant number of healthy organismoids are produced from each of these four donor vials. Blue, brown, yellow and green colors indicate each individual donor background. Subsequently, following the principles of the organismoid theory, diabetic organismoids are generated, for example, through a high glucose diet. In order to evaluate a new SGLT2 inhibitor treatment, a 3 months' trial comprising six arms is conducted with 12 organismoids per arm (three biological repeats per arm and donor) and a daily oral-like administration of the respective medication. The control arms include the healthy recovery group (1) and the diseased untreated placebo group (2) which provide the readout for cure (green baseline) and for no change of disease status (red baseline), respectively. Furthermore, three doses of the new SGLT2 inhibitor are administered at 100,000-fold lower doses than expected in patients, due to the respective downscale of organismoid size in comparison to a human body. Readouts for arms (3), (4), and (5) illustrate the potential of the new monotherapy to change the disease status from baseline toward cure at doses corresponding to 25 mg (yellow squares), 15 mg (yellow triangles), and 5 mg (yellow circles), respectively, per day and patient. A standard monotherapy arm hypothetically treated with metformin (blue circles) provides current standard care reference data. Arm (2) generates the most favorable treatment results in this hypothetical example.

The most prominent advantage is the fact that, for the first time in the history of drug development, organismoid-based trials on chips will include statistically relevant human autologous biological repeats of the patient's body and of the same individual's healthy body status. Due to the lack of any biological repeat for an individual patient and knowledge of their individual biological status at healthy homeostasis, clinical trials traditionally require large cohorts of patients. Therefore, the trials are divided into Phase 1, 2, and 3 and, unfortunately, can only approximate the pathobiology of an individual patient and their complete cure recovery status. Both aspects make the traditional clinical trial process a lengthy and incredibly costly and inefficient way of developing drugs and advanced therapies. “Clinical trials” on chips with healthy and diseased organismoids eliminate these two roadblocks. On the one hand, they allow the uniformity of inbred laboratory animal tests to be matched due to the genetic identity of each trial “participant” on an individual's organismoid level but with an entirely human background. On the other hand, the usage of organismoids of a variety of different individuals reflects the heterogeneity of patient cohorts in a clinical trial but with the advantage of statistically relevant biological repeats for each individual patient's organismoids.

Another obvious advantage of the organismoid approach is the independence from patient recruitment and hospital usage to conduct such trials. Given the existence of large PSC banks reflecting the genetic predisposition, gender and other categories relevant for the trials, an organismoid-based trial can be conducted at any time, anywhere in the world. Regarding the hypothetical example above, donor selection by diabetic predisposition, comparison of genetic ancestry and equal gender distribution might be interesting stem cell vial selection strategies.

The third advantage to mention is the flexibility regarding the trial size. The number of diseased organismoids (commonly referred to as “patients” on chips) which can be generated is theoretically unlimited. This allows the integration of pharmacokinetic aspects, finding of effective doses and combined safety and efficacy evaluation of a new chemical or biological entity in one and the same organismoid-based trial. Data which are currently generated in separate preclinical and clinical trials in laboratory animals, healthy volunteers and patients, such as the toxicity profile, the no-observed-adverse-effect-level, absorption and excretions rates, metabolite formation, the finding of effective doses, duration and scheduling of a new medication, can result from one organismoid-based trial. Our hypothetical case study to treat Type 2 diabetes could, for example, be easily extended to a larger dose range and a comparison of a single oral-like (this refers in organismoids to any administration to the apical intestine) daily administration with two doses a day. This would include a dose-dependent evaluation of efficacy while simultaneously observing the occurrence and severity of urinal or genital tract infections, well-known side effects of SGLT2 inhibitors. The definition of a therapeutic window for the use of the drug candidate in a respective patient cohort results from such an all-in-one trial, still at a preclinical candidate development stage.

Regarding both usage scenarios, we envisage that organismoids will contribute significantly to medical real-world big data collection from an individual's databases. This is due to the ability to generate unique reproducible data on microenvironmental disruption at the defined location of a first disease hit (e.g., tumor growth, virus replication) for each patient. The combination of organismoid and *in silico* approaches will further increase the predictive power for precise medications for large patient cohorts and reduce costs further.

Sophisticated *in vitro* cell culture work is usually connected in people's minds with high costs. One might speculate that the generation and processing of thousands of organismoids in a trial involves an astronomical budget because currently available MPSs are expensive both in disposable chips and operations. Here, the nature of organismoids—reflecting a self-sustainable human body—and economy of scale effects come into play. In the real world, a human body at rest can be sustained with a daily supply of about 2,000 kcal in proteins, carbohydrates and fat. This can be achieved in some poorer areas of the world for a single digit US dollar bill per person. Consequently, the daily feeding of 100,000 organismoids could be achieved for the same costs. The price of the consumable chips hosting the organismoids will predictively go down to the single dollar range as well, a downscale factor which has already been experienced with computer chips and human genome sequencing costs.

The socioeconomical dimension of the ability of organismoids to identify the best fitting medication for every individual patient and to radically cut costs and transform drug development is envisioned to be enormous. The same applies to the ethical dimension. Human MPS-based organismoids bear the potential to replace the majority of laboratory animal tests and Phase 1 and 2 clinical trials in human volunteers. They will reduce the number of Phase 3 clinical trial patients manifold. All of this will have a radically positive impact on both the patient's benefit and animal welfare on a global scale.

## Patient's Organismoids and Patient-Specific T-Cell Therapies On Chips—a Perfect Alliance to Challenge the Theory

Advanced cell therapies, such as the autologous chimeric antigen receptor (CAR) T cell therapies Kymriah^TM^ and Yescarta^TM^, have recently proven their potential to cure former treatment-resistant tumor patients (176, 177). In addition to these two CAR-T cell products approved in 2017 against hematologic tumors, several other CAR-T cell products have recently been approved. Numerous new cell therapy approaches are in the pipeline with CAR or transgenic T-cell receptors against a wide variety of tumors, infections and autoaggressive immune cells, or the use of regulatory T cells to restore immune balance in dominant undesired immune reactions (178). More than 1,000 clinical trials with immune cell products were registered worldwide at the end of 2020 (179).

This unprecedented efficacy in such areas of unmet medical need has spurred regulatory acceptance at the cost of standard safety testing procedures (180), which need to be generated retrospectively in follow-up studies of patients treated after therapy approval. That complies with the fact that a patient's response to a personalized cell therapy cannot be emulated pre-clinically in laboratory animal models because of their phylogenetic distance from the patient, the respective genotypic differences and the immunological mismatch. Similarly, a patient's responses cannot be predicted in conventional patient-derived organoid cultures, due to the lack of their integration into a systemic organismal arrangement. *Inter alia*, the emulation of the intravenous delivery of the T-cell infusion to the target site and its interaction with other major organ sites are missing crucial factors to emulate T-cell therapies and their efficacy profile in patient-derived organoids precisely. As outlined earlier, the organismoid theory here provides an alternative solution overcoming any remaining obstacles.

## What Organismoids Cannot and Should not Do

According to organismoid theory, an organismoid cannot and should not emulate the empathy or consciousness (soul or mind, respectively) which are the major parts of the sociogenesis of a human individual. Consequently, it is not able to model a patient's psychiatric disorders. The dysfunction of a 300 g human heart muscle or hip fracture and its healing rely on biophysical properties, some of which cannot be represented on organismoids due to the mismatch of scale and the physics involved.

Ethical considerations are paramount for human society and are the basis for humanity. Organismoid theory, due to its nature, introduces a number of points which must be considered ethically. Development of the human embryo until a few centimeters in scale is one of the most crucial issues. Fertilization of a human egg and its subsequent embryonic development in an artificial environment (e.g., *in vitro* culture) is prohibited in many parts of the world. The authors of the organismoid theory would like to emphasize that their ethical paradigms extend beyond this. One should not use the concepts and principles of the organismoid theory to create a human or hybrid embryo and further develop and differentiate human or hybrid tissue from that. Other methods should be used to circumvent this part of ontogenesis. The individual's consent to donate tissue to create organismoids could be a good tool to prevent misuse in the areas mentioned at an early stage.

## Conclusion

The organismoid theory presented here claims the ability to artificially recapitulate the ontogenesis of an individual's body *in vitro*, starting with a donor's stem cells and generating a defined number of identical healthy mature miniaturized body equivalents, termed organismoids, thereof. The theory further claims that such sets of donor-specific identical organismoids reflect a certain stage of that individual's healthy adulthood and can be used to simulate phases of disease and recovery relevant to that donor at a certain time in their lifespan. Modeling the individual's disease in a personalized diseased organismoid approach will provide a yet unmet realistic level of the patient's pathobiology and, consequently, provide an unprecedented tool for selecting precisely the right medicine, therapy schedule and dosing to cure the (diseased) individual.

Nature's principles of genetically and microenvironmentally encoded self-organization and maintenance of the smallest functional units of human organs and their integration into a cross talking and efficiently interacting system of blood perfusion and innervated organs are the blueprint for creating organismoids on chips. We envision them becoming the next level of emulation of human biology, providing the best possible approximation of the human counterpart. Organismoids will organically follow the organoid level of human biology *in vitro*, which, in recent years, has proven to enable the emulation of distinct functions of single tissues and organs at a miniaturized scale. Leveraging on what has been learnt from organoids, human organismoids will add the systemic innervation and supply of whole blood generated on-chip via a miniaturized physiology-based vascular and blood capillary network to the functional units of each organ equivalent. The local separation of the organotypic microenvironments of each organ equivalent from the common bloodstream by the endothelial cell layer will enable the separate organ-specific, genetically encoded and microenvironment-driven self-assembly of exact copies of the functional units of the different human organs on-chip. That, in turn, will enable the physiological cross talk of mature organ equivalents, leading to organismal on-chip homeostasis. Once established, organismoids will only require daily feeding with equivalents of digested food to emulate long-term, so-called self-sustained, body functionality on a chip.

We have illustrated that human organoid *in vitro* culture technologies and human single-organ chips produced within the last 10 years have provided vast evidence for the concept of artificial *in vitro* ontogenesis of single organ equivalents. Furthermore, human iPSC-derived multi-organ chips have furnished first indications of an accelerated artificial organ ontogenesis on chips. Finally, an ever-growing scientific literature on human disease modeling and treatment testing on human tissue chips points toward the capability of such microphysiological platforms to precisely emulate the pathobiology of a disease and the mode of action of a medicine or therapy when organismoids can be fully functionally established on MPSs. Major challenges for the further development of organ-on-a-chip systems are nervous innervation and the implementation of capillarization of the organoids, which also allows the migration of cells, especially immune cells, into the tissue.

We have enrolled the two concepts underlying the organismoid theory and detailed the principles of how to generate and use organismoids for personalized precision medicine.

The prime socioeconomic driver for challenging the organismoid theory is an urgently needed paradigm shift in advanced therapy and drug development for the much faster implementation of affordable advanced therapies and precision medicines into real-world healthcare to cure patients with unmet medical needs. The prime ethical driver is the replacement of the majority of laboratory animal tests and Phase 1 clinical trials on healthy volunteers in the drug development cycle and the shift from treating symptoms toward a curing paradigm for chronic diseases on a global healthcare level. Therefore, we have proposed accelerating the establishment of human organismoids by their first proof of concept studies in predicting the outcome, dosing and scheduling of advanced autologous T-cell therapies.

## Outlook

The MPS community envisions the first proof-of-theory for organismoids to occur within a decade, tackling the radical improvements for the healthcare system described. However, major milestones, such as the interconnection of organ equivalents by a biological vasculature and lymphatics, organ innervation, integration of microbiota, an enterohepatic circulation and, finally, a human-relevant degree of hematopoiesis need to be achieved to accomplish this aim. Above and beyond healthcare, human organismoids, once established, will provide a unique tool for the next level of basic discoveries in the life science of humans. The stable long-term functionality on such a tiny scale and the arbitrary variability in gender, age and genetic background of individual body organismoids will enable previously unimagined insights into human biology. We foresee the use of organismoids for predicting optimized diets, including the adaptation of the microbiome, on an individual or subpopulation level. The development of optimized functional synthetic food to feed the global population beyond 2,100 can be effectively guided by organismoids. The latter will serve as sensitive personalized biological sensors for environmental pollution in air and drinking water and identify potential hypersensitivity risks for their donors. Finally, they bear the potential to become the prime tool for the personalized prediction of measures to ensure the longevity of their respective donors.

## Data Availability Statement

Publicly available datasets were analyzed in this study. This data can be found here: https://www.ncbi.nlm.nih.gov/pmc/articles/PMC6745596/.

## Author Contributions

All authors listed have made a substantial, direct and intellectual contribution to the work, drafted the manuscript, revised it and approved it for publication. UM and RL developed the concept. RD, LK, and AW performed, analyzed and interpreted experiments relating to Figure 3. LK, APR, and E-MD performed, analyzed and interpreted experiments relating to Figure 4.

## Funding

Part of this work was supported by the German Federal Ministry of Education and Research (GO-Bio 3B grant agreement No. 031B0062 and Alternativmethoden – Verbund grant agreement No. 031L0099A) and the European Union's Horizon 2020 research and innovation program (RESTORE grant agreement No. 820292).

## Conflict of Interest

UM is a founder, shareholder, and the CSO of TissUse GmbH, an organ-on-a-chip company dealing with MPS-based biological models. RL is a shareholder of TissUse GmbH. APR, AW, EA, HE, LK, and E-MD are employed by TissUse. The remaining authors declare that the research was conducted in the absence of any commercial or financial relationships that could be construed as a potential conflict of interest.

## Publisher's Note

All claims expressed in this article are solely those of the authors and do not necessarily represent those of their affiliated organizations, or those of the publisher, the editors and the reviewers. Any product that may be evaluated in this article, or claim that may be made by its manufacturer, is not guaranteed or endorsed by the publisher.

## Abbreviations

3D: three-dimensional
ASCs: adult stem cells
BBB: blood–brain barrier
CAR-T: bhimeric antigen receptor T
ESCs: embryonic stem cells
hESCs: human embryonic stem cells
hiPSCs: human induced pluripotent stem cells
iPSCs: induced pluripotent stem cells
MPS: microphysiological systems
NMJs: neuromuscular junctions
PDX: patient-derived xenograft
PSCs: pluripotent stem cells
SGLT2: sodium glucose transporter 2
TEER: transepithelial electrical resistance
USD: United States dollar.
